# Susceptibility and Volume Measures of the Mammillary Bodies Between Mild Cognitively Impaired Patients and Healthy Controls

**DOI:** 10.3389/fnins.2020.572595

**Published:** 2020-09-15

**Authors:** Zhijia Jin, Sean K. Sethi, Binyin Li, Rongbiao Tang, Yufei Li, Charlie Chia-Tsong Hsu, Naying He, E. Mark Haacke, Fuhua Yan

**Affiliations:** ^1^Department of Radiology, Ruijin Hospital, Shanghai Jiao Tong University School of Medicine, Shanghai, China; ^2^Magnetic Resonance Innovations, Inc., Bingham Farms, MI, United States; ^3^Department of Radiology, Wayne State University, Detroit, MI, United States; ^4^Department of Neurology, Ruijin Hospital, Shanghai Jiao Tong University School of Medicine, Shanghai, China; ^5^Institute for Medical Imaging Technology, School of Biomedical Engineering, Shanghai Jiao Tong University, Shanghai, China; ^6^Department of Medical Imaging, Gold Coast University Hospital, Southport, QLD, Australia; ^7^Department of Biomedical Engineering, Wayne State University, Detroit, MI, United States

**Keywords:** mammillary body, quantitative susceptibility mapping, mild cognitive impairment, brain iron, volume

## Abstract

**Purpose:**

To investigate the baseline values and differences for susceptibility and volume of the mammillary bodies between mild cognitively impaired (MCI) patients and healthy controls (HCs), and further explore their differences in relation to gender, MCI subtypes and apolipoprotein E (APOE) genotypes.

**Methods:**

T1-weighted and multi-echo gradient echo imaging sequences were acquired on a 3T MR scanner to evaluate the T1W based volume and susceptibility differences in the mammillary body for 47 MCI and 47 HCs. *T*-tests were performed to compare volume and susceptibility between groups, and right and left hemispheres. Correlation analysis was used to relate the volume and mean susceptibility as a function of age in MCI and HC groups separately, and to investigate the relationship of susceptibility with the neuro-psychological scales in the MCI group.

**Results:**

Susceptibility was found to be elevated within the right mammillary body in MCI patients compared to HCs (*p* < 0.05). There were no differences for the mammillary body volumes between the MCI and HC groups, although there was a reduction in volume with age for the MCI group (*p* = 0.007). Women showed decreased mammillary body volume compared to men in the HC group (*p* = 0.004). No significant differences were found in relation to MCI subtypes and APOE genotypes. No significant correlations were observed between mammillary body susceptibility with neuro-psychological scales.

**Conclusion:**

This work provides a quantitative baseline for both the volume and susceptibility of the mammillary body which can be used for future studies of cognitive impairment patients underlying the pathology of the Papez circuit.

## Introduction

Alzheimer’s disease (AD) is the most common form of dementia which leads to cognitive impairment and memory loss. Mild cognitive impairment (MCI) was initially conceptualized as a symptomatic predementia phase of AD. The manifestation of MCI is the gradually progressive cognitive decline not yet meeting the criteria for dementia (Albert et al., 2011). Since MCI is a heterogeneous condition with different aetiologies, many studies today further classify MCI into amnestic MCI (aMCI) and non-amnestic MCI (naMCI) depending on whether memory is impaired or not (Petersen and Morris, 2005; Hughes et al., 2011). It is reported that aMCI patients have an annualized conversion rate of 12 to 28% and have 8.6-fold higher odds of developing AD (Petersen et al., 1999; Lehrner et al., 2005; Schmidtke and Hermeneit, 2008). While aMCI is highly associated with progression to AD, other subtypes of MCI patients might remain stable or even revert to normal cognition (Barnes et al., 2014; Roberts et al., 2014). Therefore, there is an increasing interest on investigating the non-invasive biomarkers underlying MCI which could potentially supplement clinical approaches for future studies.

Histopathologic hallmarks of AD include both intracellular pathological neurofibrils as well as extracellular accumulation of amyloid beta (Aβ) plaques (Selkoe, 2001; Hardy and Selkoe, 2002). Early AD has a predilection to involve the limbic system including the entorhinal cortex, hippocampus and the mammillary bodies before spreading to other brain regions (Braak et al., 1993; Xu et al., 2000; Baloyannis et al., 2016). The mammillary bodies are an essential component in Papez’ circuit, which is a network of related structures that support memory and cognition (Papez, 1995). The mammillary bodies are directly linked with brain regions that are thought to be vital for episodic memory and spatial memory (the hippocampus, anterior thalamic nuclei and tegmental regions) (Neave et al., 1997; Hildebrandt et al., 2001; Metzler-Baddeley et al., 2012). The mammillary bodies receive a dense input from the hippocampus via the fornix. Interestingly, the right and left fornical fibers convey different functional information. The left fornix primarily conveys verbal memory while the right conveys visuospatial memory (Tucker et al., 1988; Hodges and Carpenter, 1991; McMackin et al., 1995; Saunders and Aggleton, 2007). This connection continues from the mammillary bodies to the anterior thalamic nuclei by way of the mammillothalamic tract (Cruce, 1975; Vann et al., 2007, 2011). Earlier hypotheses only considered the mammillary bodies as a hippocampal relay, which underestimates their independent role in memory. Actually, except for this circuit, it has been assumed that the reciprocal connections between the mammillary bodies and tegmental regions (Bassett and Taube, 2001) are independent of the hippocampus but are critical for sustaining the mammillary bodies’ function in memory (Vann, 2009). Damage to the mammillary bodies and their connection fibers can lead to an inability to lay down new episodic memories (Tsivilis et al., 2008; Metzler-Baddeley et al., 2012). However, few imaging studies have focused on the functionality of the mammillary bodies in humans due to their location and small size.

In addition to Aβ plaques and neurofibrillary degeneration, elevated brain iron and CSF ferritin are increasingly reported as a hallmark of AD that could perhaps predict MCI conversion to AD (Ayton et al., 2015, 2017; Kim et al., 2017). The specific underlying mechanisms of iron accumulation in AD remain unclear. Nevertheless, the close link between iron and the pathogenesis of AD is supported by a variety of evidence. For example, it has been observed that iron accumulates in the same brain regions that exhibit Aβ accumulation, such as the hippocampus, frontal cortex and parietal cortex, and most amyloid plaques are found to contain iron (Good et al., 1992; Liu et al., 2011). Iron accumulation is also associated with neuro-fibrillary tangle (NFT) formation, and the binding of ferric iron and tau protein is found to precede the accumulation of hyperphosphorylated tau and the subsequent formation of NFTs (Smith et al., 1997; Yamamoto et al., 2002; Li et al., 2015). The redox-active iron combined with the Aβ and NFT aggregation in AD generate increasing oxidative stress. Under such circumstances, the visualization and reliable quantitative evaluation of excess iron accumulation suggests the potential theraputic benefit of lowering brain iron in AD patients.

Several studies using T_2_^∗^ mapping and susceptibility weighted imaging (SWI) found increased iron content in brain gray matter including the hippocampus, frontal and parietal regions in AD patients (Wang et al., 2013; van Rooden et al., 2014). However, conventional gradient echo (GRE) imaging approaches are affected by blooming artifacts and cannot be easily quantified. Phase images do not directly measure local tissue susceptibility due to the non-local relationship between phase and the underlying magnetic susceptibility distribution (Schweser et al., 2011; Li et al., 2012). Quantitative susceptibility mapping (QSM) has made it possible to directly map tissue magnetic susceptibility which has been shown to correlate well with iron content in brain tissue (Liu et al., 2009; Langkammer et al., 2012; Lim et al., 2013).

Therefore, the goal of this study was to generate susceptibility and volume baseline values as a function of age for the mammillary bodies to ascertain if there is an iron content increase and to investigate the susceptibility and volume differences of the mammillary bodies between healthy controls (HCs) and MCI patients. In addition, we also investigated the susceptibility and volume differences of the mammillary bodies between genders, aMCI, naMCI and MCI patients with and without apolipoprotein E (APOE) ε4.

## Materials and Methods

### Participants

This study was approved by the local ethics committee and all subjects signed a consent form. Forty-seven (47) MCI subjects (mean age: 63.8 ± 6.9 years, range: 51–78 years; 33 females) were recruited from the memory clinic of the Neurology Department in Ruijin Hospital. Forty-seven (47) age- and gender-matched HCs (mean age: 63.5 ± 6.6 years, range: 51–75 years, 33 females) were recruited from the local community by advertisement. All participants were of unrelated Chinese Han descent with >6 years of education (MCI: 12.3 ± 2.5 years, HC: 11.2 ± 3.8 years). According to the diagnostic guidelines of the United States National Institute on Aging-Alzheimer’s Association (NIA-AA) (Albert et al., 2011) for MCI due to AD, our diagnosis was based on a detailed medical history interview, neurologic examinations and cognition tests. All MCI patients were screened using the Mini Mental State Examination (MMSE, 24 ≤ MMSE ≤ 28) (Katzman et al., 1988), the Hamilton Depression Rating Scale (HAMD score ≤ 10) (Hamilton, 1960) and the global score of Clinical Dementia Rating Scale (global score = 0.5) (Morris, 1997). Neuro-psychological assessment for the episodic memory and visuospatial memory using the Auditory Verbal Learning Test (AVLT)-Huashan version (Zhao et al., 2012) and the Rey–Osterrieth Complex Figure Test (CFT) (Shin et al., 2006) was performed by two neurologists with 3 years of experience each. Patients with MCI were further classified into two subtypes: aMCI, if the number of words answered correctly in the 20-min delayed recall of the AVLT (AVL-LR) was less than 4, or naMCI, if the AVL-LR number was greater than 4 (Zhao et al., 2012). The APOE genotype of 45 MCI patients was measured from peripheral blood samples, with 2 of the MCI patients missing the blood sample collection. None of the HCs had memory complaints or indications of cognitive deficits. The exclusion criteria for both the MCI and HC groups included the following: (a) structural abnormalities that could impair cognitive function, such as tumor, subdural hematoma, or contusion from a previous head trauma; (b) a history of stroke, addiction, neurologic or psychiatric disease, or treatment that would affect cognitive function; and (c) large-vessel disease and/or diseases with large volume white matter lesions (i.e., Fazekas grade III).

### Image Acquisition

All subjects were imaged with gradient echo and T1-weighted (T1W) imaging sequences on a 3T Ingenia scanner (Philips Medical Systems, Best, The Netherlands) with a fifteen-channel phased array head coil. The three-dimensional (3D) multi-echo GRE data were acquired with the following imaging parameters: TR = 59.3 ms, number of echoes = 16, TE1 = 2.7 ms, TE spacing = 2.9 ms, bandwidth = 488 Hz/pixel, flip angle = 12°, field of view (FOV) = 22 cm, matrix size (Nx × Ny) = 256 × 256, resolution = 0.86 × 0.86 × 1.0 mm^3^, acceleration factor = 2 (approximately 70% of k-space was sampled), slices = 136 and total acquisition time = 10 min 42 s. The sagittal 3D T1W images were acquired with a magnetization-prepared rapid acquisition gradient echo (MPRAGE) sequence with the following parameters: TR = 8.1 ms, TE = 3.7 ms, flip angle = 8°, FOV = 236 mm × 236 mm, and an acquisition voxel size = 1 mmł isotropic. Conventional MR images, including 2D T2-weighted fluid-attenuated inversion recovery (FLAIR) and 2D diffusion weighted imaging (DWI), were acquired in the transverse plane for screening of cerebrovascular diseases, white matter hyperintensity and space-occupying lesions in the brain. The whole brain was covered for all MR scans, including the multi-echo GRE sequence. All transverse oriented scans were collected along the anterior commissure–posterior commissure (AC-PC) line.

### Image Reconstruction and Post-processing

**FIGURE 1:**
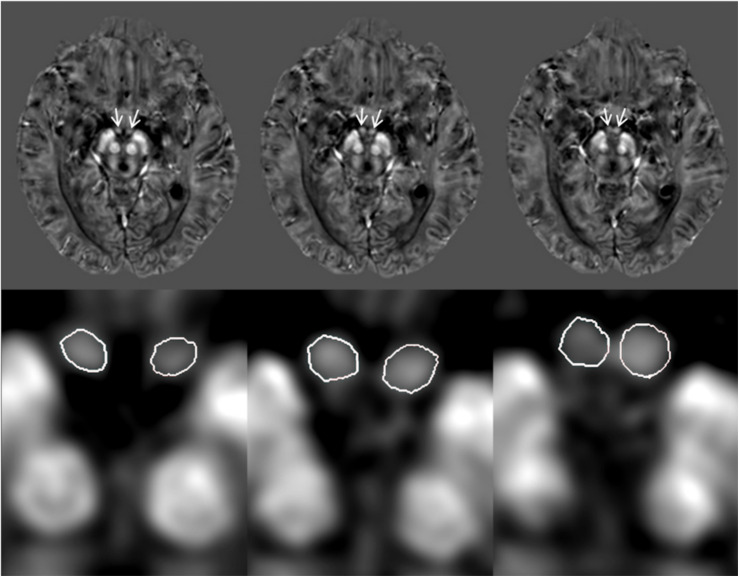
Quantitative susceptibility maps (QSM) showing example tracings of the mammillary bodies for a subject with mild cognitive impairment (MCI). The first row: original QSM images, arrows pointing to the mammillary bodies. The second row: 8X zoom of images corresponding with the first row images showing the traced mammillary bodies.

A custom algorithm was used to generate QSM using data from all 16 echoes (Li et al., 2011). All QSM and T1W data were traced on an interpolated zoomed image (8X magnification) using SPIN Software (SpinTech, Inc., Bingham Farms, Michigan, United States). A semi-automated dynamic programming method was used to demarcate the right and left mammillary bodies (Jiang et al., 2007). An intra-class correlation coefficient (ICC) statistic for absolute agreement (Shrout and Fleiss, 1979) was calculated between two raters who traced the mammillary bodies on the QSM and T1W images for 30 randomized cases (15 HC and 15 MCI). An example of the boundary delineation on the QSM data is illustrated in Figure 1. The mammillary bodies were visible on 3–4 consecutive slices on the QSM and T1W images.

### Statistical Analysis

An independent two-sample *t*-test was performed to compare age and education between the MCI and HC groups. A chi-square test was performed to assess the inter-group gender heterogeneity between these groups. Independent-sample *t*-tests were performed to compare volume and susceptibility in the mammillary body between patients with MCI and HCs, patients with aMCI and naMCI, APOE ε4 carriers and non-carriers, and female and male. To observe whether bilateral differences of volume and susceptibility exist within the mammillary bodies in MCI and HC subjects, paired-sample *t*-tests were performed. For the *t*-tests, a value of *p* < 0.05 was considered significant. Pearson correlation analyses were used with age as an independent variable using volume and mean susceptibility as dependent variables in MCI and HC groups. To investigate the correlation of mean susceptibility in the mammillary bodies with the AVLT and CFT scores, a partial correlation analysis was performed with age as a covariate. A conservative statistical significance of *p* value < 0.01 was used in the multiple correlation analysis to control for the inflation of type I error. All statistical analyses were run on the data using SPSS (IBM Corp. Released 2013. IBM SPSS Statistics for Windows, Version 22.0. Armonk, NY: IBM Corp.).

## Results

### Demographic and Clinical Characteristics Analyses

There was no significant difference of age, gender or education between the MCI and HC groups. The MMSE score for MCI patients was 27.5 ± 0.7. For the episodic memory and visuospatial memory assessments, the AVLT scores were 15.6 ± 4.7 for immediate recall, 4.6 ± 2.3 for 5-min recall, 4.6 ± 2.4 for 20-min recall and 19.9 ± 2.7 for 20-min recognition. The CFT scores were 35.3 ± 1.6 for copy and 16.9 ± 7.7 for recall. According to the 20-min delayed recall of the AVLT, 22/47 MCI patients were classified into the aMCI group and 25/47 were put in the naMCI group. Further, 45 MCI patients with the APOE genotype were also broken up into a high-risk group with APOE ε4 (*n* = 15) and a low-risk group without APOE ε4 (*n* = 30).

### Susceptibility Analysis

**FIGURE 2:**
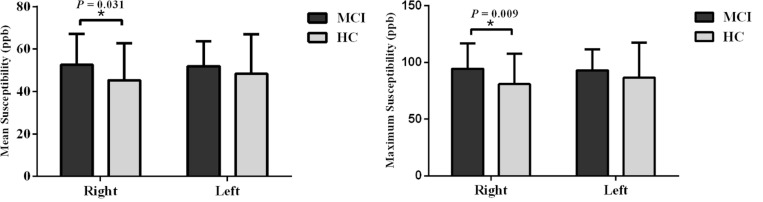
Group analyses for the mean susceptibility and maximum susceptibility in the mammillary body between the MCI and HC groups. Significant differences between MCI and HC subjects are represented as: *p < 0.05. MCI: mild cognitive impairment, HC: healthy control.

**FIGURE 3:**
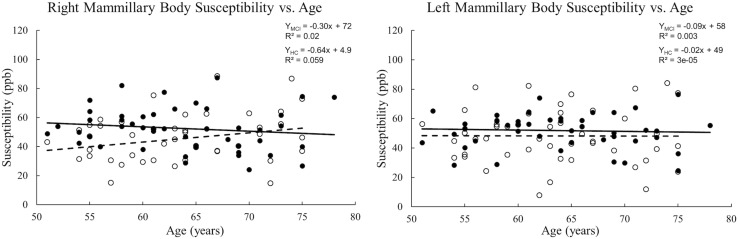
Scatter plots and regression lines for mean susceptibility vs. age for 47 MCI patients and 47 HC subjects. Solid circles: MCI, Open circles: HC. Dotted line: HC, Solid line: MCI. The **right** and **left** mammillary bodies are plotted separately. There is no significant change in mean susceptibility with age in either group.

When tracing the mammillary bodies on QSM using the semi-automated method, the ICCs for the right and the left sides were 0.91 and 0.92, respectively. A breakdown of the mean susceptibility, maximum susceptibility, and statistics for paired *t*-tests between right and left sides is shown in Supplementary Table S1. No differences were observed between sides for any of the groups (all *p* values > 0.05). As shown in Figure 2, mean susceptibility and maximum susceptibility increased in the right mammillary body in the MCI group as compared to HC group, but no significant susceptibility difference was found in the left mammillary body. There was no significant change of mean susceptibility as a function of age in the right or left mammillary bodies in either the MCI group, or the HC group as shown in Figure 3. No significant susceptibility differences in the mammillary body were found in relation to gender, MCI subtypes or APOE genotypes (Supplementary Table S2). No significant correlations (*p* > 0.01) were observed between mean susceptibility of the mammillary body and 5-min delayed AVLT score, 20-min delayed AVLT score, or the CFT recall score in the MCI group.

### T1W Based Volume Analysis

**FIGURE 4:**
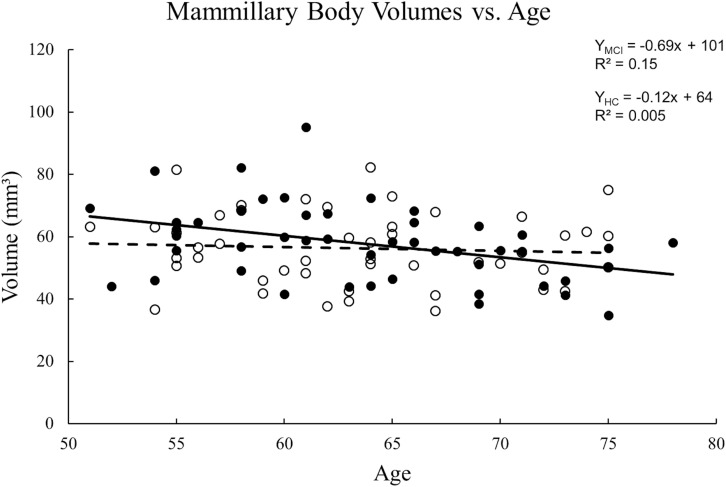
Scatter plots and regression lines for the average mammillary body volume vs. age for 47 MCI patients and 47 HC subjects. Solid circles: MCI, Open circles: HC. Dotted line: HC, Solid line: MCI. There is a significant reduction in volume with age for MCI patients but not the HCs.

The ICC between the two raters for tracing the mammillary body volumes on T1W was 0.85. No volume differences were observed between MCI and HC groups in either sides of the mammillary body (*p* > 0.05). Women showed significant decreased mammillary body volume when compared to men in the HC group, but not in the MCI group (Supplementary Table S2). No significant volume differences were found in the mammillary body between aMCI and naMCI, or APOE ε4 carriers and non-carriers (Supplementary Table S2). There was no volume significance between right and left sides of the mammillary body in MCI and HC groups (*p* > 0.05), thus we used the average volume of both sides for correlation analysis. A negative correlation between volume and age (Figure 4) was observed in the MCI group (R^2^ = 0.152, *p* = 0.007), while in the HC group, no significant correlation was found (*p* > 0.01).

## Discussion

The mammillary bodies and their main projections play a substantial role in spatial memory and delayed memory recall. This is the motivation to investigate the baseline values and differences for susceptibility and volume of the mammillary bodies between MCI and HC subjects. To our knowledge, this is the first study using QSM to quantify iron deposition in the mammillary bodies in MCI. The results showed a susceptibility roughly equal to that of gray matter, around 50ppb, with an increased iron level in the right mammillary body in MCI patients compared with HCs. The susceptibility values for the mammillary bodies presented herein could be used as a baseline for future studies of brain iron change in cognitively impaired patients including patients with AD, behavioral variant frontotemporal dementia (FTD) and amyotrophic lateral sclerosis (ALS) which affect the Papez circuit.

*In vivo* studies of the iron accumulation in the mammillary bodies are limited (De Reuck et al., 2014). A previous magnetic resonance imaging study at 7.0 T of iron deposition in post-mortem brains of patients with neurodegenerative and cerebrovascular diseases observed no iron accumulation in the mammillary bodies (De Reuck et al., 2014). However, in that study, the severity of the iron load was evaluated semi-quantitatively using a ranking approach based on the intensity and distribution of hypo-intensity signal on T2^∗^-weighted imaging. Generally, T2^∗^ is not as sensitive to iron changes as QSM and is also a noisier measurement. Both post-mortem experiments (Langkammer et al., 2012; Sun et al., 2015) and *in vivo* (Schweser et al., 2011; Bilgic et al., 2012) experiments have demonstrated that magnetic susceptibility assessed by QSM is highly correlated with known iron concentrations of various structures in the brain. The iron levels measured here of roughly 50 ppb are similar to those seen between gray matter and white matter and are sufficiently higher than the noise level to be clearly visible on the QSM data. Therefore, our approach using QSM to quantify the iron level is a reasonable approach to detect iron changes of the mammillary bodies in the progression of cognitive decline. We also explored the mean susceptibility of the mammillary body as a function of age in both the MCI group and the HC group. However, no significant correlations between susceptibility and age were discovered.

In this study, a significant reduction in volume as a function of age was found in MCI patients but there was no statistical difference in volumes between MCI patients and HCs. The latter finding is consistent with previous research (Copenhaver et al., 2006) showing no significant volume differences between HC and MCI groups. Nevertheless, they found significant volume reductions in the mammillary bodies in patients with AD as compared with HC and MCI participants, which suggested that atrophy of the mammillary bodies became obvious at the point of conversion from MCI to AD (Copenhaver et al., 2006). Our findings confirmed the relative preservation of the mammillary bodies volume in early disease stages.

In addition, a decreased mammillary body volume in women compared to men in the HC group was found in this study. Interestingly, Falcon et al. found a greater atrophy in medial temporal areas (Papez circuit) associated to CSF p-tau and glial biomarkers in cognitively unimpaired women compared to men (Falcon et al., 2020). A greater atrophy of the structures in the Papez circuit associated with baseline CSF p-tau may partially explain women’s greater susceptibility to memory complaints and AD pathology. Although a lot of evidence suggests a higher incidence of AD in women, it is still not clear whether the greater prevalence is due to their longer life expectancy or other underlying factors (Beam et al., 2018; Medeiros and Silva, 2019). In this study, we did not find volume or susceptibility differences in the mammillary body between women and men in the MCI patients, although some studies found greater medial temporal atrophy in early stages of AD in women (Apostolova et al., 2006; Hua et al., 2010; Ardekani et al., 2016). The inability of detecting the gender differences in the mammillary body in our study could be partly due to the heterogeneous conditions of MCI with different aetiologies and the relatively small sample size. Further classification of the MCI cohort showed no susceptibility or volume differences in the mammillary body between aMCI and naMCI groups. It may be interesting to further investigate the gender differences of the mammillary body in aMCI patients with a larger sample size in the future.

The APOE ε4 allele is recognized as the main genetic risk factor for AD and MCI as well (Kryscio et al., 2006). Previous studies showed inconsistent results of volumetric analyses of APOE ε4 carriers focused on medial temporal lobe with some reported reduced hippocampal volumes (O’Dwyer et al., 2012), and others not (Adamson et al., 2010; Li et al., 2014). Our exploration of APOE genotypes found no susceptibility or volume differences in the mammillary body between APOE ε4 carriers and non-carriers. In addition to the volume studies, functional connectivity analyses of the Papez circuit with the hippocampus as a ‘seed’ found that APOE ε4 carriers had significantly decreased functional connectivity in brain areas such as the thalamus, caudate nucleus, and cingulate cortices involved in the Papez circuit (Li et al., 2014). They also found that the decreased hippocampal functional connectivity in these regions were positively correlated with the episodic memory performance (Li et al., 2014). These findings illustrate the importance of the integrity of the Papez circuit on episodic memory.

Damage to the integrity of gray matter and their connection fibers in the Papez circuit can lead to cognition impairment in various neurodegenerative diseases including AD, behavioral variant FTD and ALS (Thomas et al., 2011). Both *in vivo* and postmortem studies suggest that subregions of the Papez circuit are differentially affected in behavioral variant FTD and AD (Hornberger et al., 2012; Bertoux et al., 2014), with behavioral variant FTD patients having more severe neural degeneration of the fornix, anterior thalamus and anterior cingulate cortex and AD patients having more significant atrophy of the posterior cingulate cortex. Clinically mild psychiatric and cognitive impairment of patients with ALS before the onset of motor symptoms can cause a concomitant diagnose of behavioral variant FTD due to their overlap of clinical, pathological and genetic characteristics (Salameh et al., 2015). Therefore, more studies have focused on the investigation of the non-invasive biomarkers for the differentital diagnosis. It has been found that the entorhinal cortex and mammillary body atrophy may help to distinguish behavioral variant FTD from ALS with behavioral variant FTD patients showing more atrophy than patients with ALS (Bueno et al., 2020). In addition, Bueno et al. (2018) showed widespread structural and functional connectivity abnormalities across the structures composed of the Papez circuit, while the thalamus, mammillary bodies and fornix were preserved in ALS patients. The post-mortem study also found substantial atrophy of the mammillary bodies and anterior thalamus in behavioral variant FTD (Hornberger et al., 2012). All in all, the integrity of the Papez circuit and its entire subregions should be taken into consideration when establishing whether the memory loss observed in a patient is likely to be due to AD or other neurodegenerative diseases such as behavioral variant FTD and ALS.

Drawing strong conclusions about the iron and volume changes of the mammillary bodies in MCI subjects will require evaluating a much larger population. It would be particularly interesting to follow longitudinal changes for a given individual. As with all *in vivo* MRI studies of MCI, our study is limited by the lack of pathological confirmation of the disease state. Nevertheless, at our institute all MCI patients will eventually undergo longitudinal clinical evaluation with reassessment of their diagnosis over time. Longitudinal assessments of the volumetric reduction may be useful in determining the conversion point from MCI to AD.

## Conclusion

MCI paitents showed increased susceptibility in the right mammillary body compared to HCs. The structural volume of the mammillary bodies decreased with age in MCI patients. Women showed decreased mammillary body volume than men in the HCs. This work provides an important baseline for studying the mammillary bodies in cognitive impairment patients not only in AD but also for other neurodegenerative diseases such as FTD and ALS which affect the Papez circuit.

## Data Availability Statement

The raw data supporting the conclusions of this article will be made available by the authors, without undue reservation.

## Ethics Statement

The studies involving human participants were reviewed and approved by Ruijin Hospital, Shanghai Jiao Tong University School of Medicine, Shanghai, China. The patients/participants provided their written informed consent to participate in this study.

## Author Contributions

EMH originally conceived of the method. ZJ and BL contributed to the data acquisition. EMH, SS, ZJ, and YL contributed to the analysis and interpretation of the data. ZJ and SS drafted the manuscript. All authors contributed to the critical revision and final presentation of the work.

## Conflict of Interest

SS and EMH were employed by the company Magnetic Resonance Innovations, Inc. The remaining authors declare that the research was conducted in the absence of any commercial or financial relationships that could be construed as a potential conflict of interest.

## Supplementary Material

TABLE S1 The T1W based volume, mean susceptibility and maximum susceptibility are presented, along with paired *t*-test results between the left mammillary body and right mammillary body for MCI patients and HCs.

TABLE S2 Independent-sample *t*-test analyses between genders, aMCI and naMCI patients, MCI patients with APOE ε4 and without APOE ε4 for total volume and mean susceptibility in the mammillary body.
