# Supplementary material for: Susceptibility and Volume Measures of the Mammillary Bodies Between Mild Cognitively Impaired Patients and Healthy Controls
Source: Front Neurosci. 2020 Sep 15;14:572595. doi: 10.3389/fnins.2020.572595 (PMC7522522; doi:10.3389/fnins.2020.572595)
Supplement: TABLE S1 — The T1W based volume, mean susceptibility and maximum susceptibility are presented, along with paired t-test results between the left mammillary body and right mammillary body for MCI patients and HCs. [file Table_1.DOCX]

**Supplementary Table 1:** The T1W based volume, mean susceptibility and maximum susceptibility are presented, along with paired t-test results between the left mammillary body and right mammillary body for MCI patients and HCs.

|  | | | | | | |
| --- | --- | --- | --- | --- | --- | --- |
| **Group** |  | **Measure** | **Mean** |  | **Std** | **p** |
| **MCI** |  | **Left Volume (mm³)** | 57.4 |  | 13.4 | 0.49 |
|  |  | **Right Volume (mm³)** | 58.1 |  | 12.1 |  |
|  |  | **Left Mean Susceptibility (ppb)** | 51.9 |  | 11.8 | 0.72 |
|  |  | **Right Mean Susceptibility (ppb)** | 52.6 |  | 14.6 |  |
|  |  | **Left Maximum Susceptibility (ppb)** | 93.1 |  | 18.32 | 0.66 |
|  |  | **Right Maximum Susceptibility (ppb)** | 94.4 |  | 22.45 |  |
| **HC** |  | **Left Volume (mm³)** | 56.5 |  | 11.5 | 0.63 |
|  |  | **Right Volume (mm³)** | 56.1 |  | 12.4 |  |
|  |  | **Left Mean Susceptibility (ppb)** | 48.3 |  | 18.6 | 0.19 |
|  |  | **Right Mean Susceptibility (ppb)** | 45.3 |  | 17.4 |  |
|  |  | **Left Maximum Susceptibility (ppb)** | 86.5 |  | 31.1 | 0.15 |
|  |  | **Right Maximum Susceptibility (ppb)** | 80.9 |  | 26.9 |  |
