# Supplementary material for: Susceptibility and Volume Measures of the Mammillary Bodies Between Mild Cognitively Impaired Patients and Healthy Controls
Source: Front Neurosci. 2020 Sep 15;14:572595. doi: 10.3389/fnins.2020.572595 (PMC7522522; doi:10.3389/fnins.2020.572595)
Supplement: TABLE S2 — Independent-sample t-test analyses between genders, aMCI and naMCI patients, MCI patients with APOE ε4 and without APOE ε4 for total volume and mean susceptibility in the mammillary body. [file Table_2.docx]

**Supplementary Table 2:** Independent-sample t-test analyses between genders, aMCI and naMCI patients, MCI patients with APOE ε4 and without APOE ε4 for total volume and mean susceptibility in the mammillary body.

|  |  | | | | | |
| --- | --- | --- | --- | --- | --- | --- |
| **Group** | | **Measure** | **t** | **df** | **P** |  |
| **HC (female vs. male)** | | **Total Volume (mm³)** | 3.062 | 45 | **0.004** |  |
|  | | **Right Mean Susceptibility (ppb)** | 0.204 | 45 | 0.839 |  |
|  | | **Left Mean Susceptibility (ppb)** | -1.026 | 45 | 0.311 |  |
| **MCI (female vs. male)** | | **Total Volume (mm³)** | 0.653 | 45 | 0.517 |  |
|  | | **Right Mean Susceptibility (ppb)** | -0.736 | 45 | 0.175 |  |
|  | | **Left Mean Susceptibility (ppb)** | -1.160 | 18 | 0.261 |  |
| **aMCI vs. naMCI** | | **Total Volume (mm³)** | 0.032 | 41 | 0.974 |  |
|  | | **Right Mean Susceptibility (ppb)** | -0.28 | 45 | 0.781 |  |
|  | | **Left Mean Susceptibility (ppb)** | -0.862 | 45 | 0.393 |  |
| **APOE ε4 (carrier vs. noncarrier)** | | **Total Volume (mm³)** | 0.161 | 43 | 0.873 |  |
|  | | **Right Mean Susceptibility (ppb)** | -0.399 | 43 | 0.692 |  |
|  | | **Left Mean Susceptibility (ppb)** | -0.378 | 43 | 0.707 |  |

Bold, P<0.05, significantly different between groups.

HC: healthy control; aMCI: amnestic mild cognitive impairment; naMCI: non-amnestic mild cognitive impairment.
